# From First to Second: How Stickler’s Diagnostic Genetics Has Evolved to Match Sequencing Technologies

**DOI:** 10.3390/genes13071123

**Published:** 2022-06-23

**Authors:** Howard Martin, Allan J. Richards, Martin P. Snead

**Affiliations:** Stickler’s Higher Specialist Service, Addenbrooke’s Hospital, Hills Road, Cambridge CB2 0PY, UK; ar204@medschl.cam.ac.uk (A.J.R.); martin.snead@addenbrookes.nhs.uk (M.P.S.)

**Keywords:** next-generation sequencing, diagnostic genetics, NHS

## Abstract

Diagnostic genetics within the United Kingdom National Health Service (NHS) has undergone many stepwise improvements in technology since the completion of the human genome project in 2003. Although Sanger sequencing has remained a cornerstone of the diagnostic sequencing arena, the human genome reference sequence has enabled next-generation sequencing (more accurately named ‘second-generation sequencing’), to rapidly surpass it in scale and potential. This mini review discusses such developments from the viewpoint of the Stickler’s higher specialist service, detailing the considerations and improvements to diagnostic sequencing implemented since 2003.

## 1. Background

A primary shift in diagnostic genetics resulted from the 2003 UK Government’s white paper ‘Our Inheritance, Our Future—Realizing the potential of genetics in the NHS’. It marked both the 50th anniversary of Crick and Watson’s discovery of the double helix structure of DNA, and the completion of the human genome project, and served as a starting point that has seen the expansion and standardization of diagnostic genetic testing in the NHS. 

At that point, diagnostic testing for approximately 200 genetic disorders was routinely available in the NHS; however, many genes were still only partially sequenced due to cost and processing constraints. 

The subsequent investment in equipment and techniques enabled the NHS to expand both its genetic testing portfolio and the speed of diagnostic testing. For Stickler’s syndrome, this enabled the full exon content of pertinent collagen genes to be simultaneously sequenced at scale, for the first time (UKGTN, https://digital.nhs.uk/, accessed on 18 June 2022).

## 2. Key Considerations in First-Generation Sequencing

First-generation sequencing (Sanger sequencing, or capillary sequencing) relies heavily on polymerase chain reactions (PCRs) to isolate and sequence target genes. In its simplest form, PCR uses a pair of primers as the basis to amplify a DNA region bounded by the primer binding locations. Considered primer designs can significantly affect PCR performance, as can the sequence of the region being amplified. Few genes maintain an optimal percentage of guanosine and cytosine bases (% GC) throughout their length, compounding amplification by Taq polymerase. Especially challenging are regions with suboptimal % GC, regions with repeating sequence motifs, regions of homonucleotide repeats [1], and sequences with significant self-homology and stable secondary structure, all of which compound the ability of DNA polymerase to accurately copy a DNA template. The development of a single PCR optimal for all regions of a gene is, consequently, rare to achieve.

These issues constrain the optimization of amplifications required to generate full gene coverage, leading to multiple PCR conditions and reaction mixes. For Stickler’s syndrome diagnosis, dozens of differing amplification conditions are required to enable testing of a single gene, resulting in an exon-based batching approach where identical gene regions were simultaneously amplified in multiple patient samples.

To achieve the throughput necessary to rapidly test individual patients, gene amplification reactions should ideally be performed simultaneously under the same amplification conditions to generate PCR products that tile across the target gene.

In the absence of a single PCR buffer system suitable for all amplifications, 12 separate PCR buffers covering a range of magnesium chloride and betaine concentrations were used for Stickler’s diagnosis (MasterAmp^TM^, CamBio, Cambridge, UK).

This enabled primers and buffers to be pre-mixed in a 96-well plate, acting as a master plate sufficient for 10 patient samples to be processed. This enabled all amplifications to be performed in a single plate significantly reduced turnaround time for patient testing (Appendix A).

Chemistry and equipment for first-generation sequencing focused on offerings from Applied Biosystems (ABI, now Thermo Fisher Scientific, Waltham, MA, USA), with capacity available to sequence up to 96 samples simultaneously. For the Stickler’s diagnostic service in the early 2000s, this consisted of BigDye v1.1 chemistry and the ABI 3130xl capillary sequencer (Thermo Fisher Scientific, Waltham, MA, USA).

## 3. First-Generation Sequencing of Stickler’s Syndrome

To process individual amplicons into sequence-ready material, a tagged primer approach was developed. M13 bacteriophage sequences were incorporated into the amplification primers such that these sequences could be used to initiate the sequencing of all amplicon targets, irrespective of the initial amplification primers used.

To screen Stickler’s syndrome genes, a unidirectional sequence strategy was used, requiring careful selection of the sequencing orientation and avoiding regions causing issues in sequence generation. Although such regions could be determined experimentally, manual examination of the gene sequence could determine regions of issue. Consequently, overlapping sequence could be generated to enable the full gene to be tested, albeit with sequence reads in various orientations. Although this determined the gene sequence, a significant concern in this approach was the confidence that both genomic alleles were being sequenced. Single nucleotide polymorphisms (SNPs) under the primer binding sites have the potential to cause allele dropout during amplification in both first-generation sequencing and current second-generation sequencing approaches [2]. To reduce the risk of such dropout, primer design required a consistent SNP checking approach, using software developed by the National Genetics Reference Laboratory (NGRL, Manchester, UK), to identify validated SNPs from the dbSNP database (https://genetools.org/SNPCheck/snpcheck.htm, accessed on 1 January 2022 (https://www.ncbi.nlm.nih.gov/snp/, accessed on 1 January 2022).

## 4. The Non-Quantitative Nature of Amplification and Sequencing

Despite controls in primer design, reliably identifying quantitative changes in gene copy number requires a separate non-sequencing-based approach. To detect genomic deletion and duplication rearrangements events below the resolution of conventional karyotyping (approx. 1–2 Mb), applicable techniques included multiplex amplifiable probe hybridization (MAPH, [3]) multiplex ligation-dependent probe amplification (MLPA, MRC Holland, [4]) and multiplexed quantitative PCR. Of these, MLPA proved most suited to diagnostic use, consisting of DNA probe hybridization and the ligation of adjacent probes, generating a template that can be quantitatively amplified and resolved using capillary electrophoresis. For diagnostic copy number detection in Stickler’s syndrome, probe mixes for COL11A1 (P381 and P382) and COL2A1 (P214) were utilized.

## 5. Diagnostic First-Generation Sequence Data Analyses

Although improvements in sequencing chemistry and equipment have been the main drivers in diagnostic genetics, such developments require evaluation for the diagnostic community: for batch analysis of capillary sequence data, the majority of diagnostic laboratories chose Mutation Surveyor software (Softgenetics, PA, USA). Software, as well as reagents and equipment, require thorough validation prior to use in a diagnostic setting, because the overwhelming majority of such products are supplied for ‘research use only’, with the onus on the diagnostic community to validate and establish performance metrics necessary to enable the confident reporting of test results using such products.

## 6. Comparison of First- and Second-Generation Sequencing Approaches

First-generation capillary sequencing uses a single primer per template to generate dye-labelled copies of the DNA, with individual bases becoming tagged with fluorescent labels. These copies are separated on the basis of size using a capillary tube filled with a polymer across which a high voltage is applied. The visual detection of the dye-linked bases is achieved using a powerful laser to induce fluorescence, with the colour detected corresponding to the fluorescent base being interrogated, reviewed in [5,6]. This approach necessitates that all target molecules are sequenced from the same starting location to produce a consensus sequence result.

A key advantage to this approach is the ability to generate long reads of sequence up to 800 bases in length, with quality scores assigned on a per base basis: The size of the template from which this sequence is derived can be significantly larger than the final length of sequence obtained. With second-generation sequencing, however, the template size must be matched to the smaller length of sequence read that can be obtained, with a maximum read of 300 bases currently achievable using Illumina technology, enabling 600 bases of sequence to be obtained if the template is sequenced from both ends consecutively, a technique described as paired-end sequencing (Illumina, San Diego, CA, USA).

The sequence data determined by capillary sequencing represents the summed population of templates in a single output; therefore, mosaicism detection and the phasing of variants is not feasible without further bespoke testing. Low-level mosaicism, below 5% of the template molecules within the template pool, is problematic to detect. Visual examination remains the most successful approach to determine such instances, compounding the detection of pathogenic variants in a number of diseases where mosaicism is prominent, e.g., tuberous sclerosis [7].

Second-generation sequencing, conversely, allows each individual template molecule to generate a sequence which is assembled to produce a consensus sequence of the original templates. This enables individually sequenced templates to be examined for the phasing of variants. Second-generation sequencing has a defined number of sequence reads that can be generated per run; thus, controlling the relative abundance of a template in the pool of templates enables more sequence reads to be generated from such templates. This increases both the number of reads that can detect a mosaic variant and the confidence of correctly identifying the variant [8], affording significant improvements in the detection of minor allele variants in constitutional genetic disease, tumour tissue [9], and viral populations such as HIV [10].

Clinical diagnostic laboratories process large numbers of patient samples and templates for sequencing; therefore, the use of high-throughput liquid handling automation and sample batching approaches are required to maximize processing throughput. However, the advent of second-generation sequencing enabled a significant improvement in the scale of sequencing that could be obtained beyond that available with automated sample processing.

Although PCR remains central to second-generation sequencing, the approach to generating templates for the sequencing reaction differs significantly. With first-generation sequencing, a single primer binds all templates at the same location, and progressively sequences the template as it is being replicated. In second-generation sequencing, this primer binding occurs in an exogenous portion of DNA that has been added to the terminus of the DNA template, akin to the M13-tagged template approach. These exogenous DNA adapters are added to a large population of templates in a sequence-independent manner, with each template representing a different region of the gene under analysis. Consequently, in second-generation sequencing, one has a diverse pool of templates being sequenced with a single primer, but each template within the pool generates unique sequence data. Were this to be separated using a capillary electrophoresis machine, it would be impossible to separate individual template molecules, and individual sequences. In order to discern individual sequences from a pool of templates, each template molecule must be tethered to a solid support surface (usually a glass slide), where the physical location of the sequenced template remains unchanged during the sequencing reaction. In effect, a glass slide now replaces the 96-well plate approach used in capillary electrophoresis sequencing, as reviewed in [11].

This ability to sequence a template molecule in isolation within a heterogeneous pool has led to a number of new approaches for generating such templates. These include highly multiplexed PCR amplification (AmpliSeq, Thermo Fisher, Waltham, MA, USA) and genome fragmentation and enrichment-based techniques, as reviewed in [12].

This has made feasible the sequencing of many thousands of template molecules simultaneously, hence the description of second-generation sequencing as ‘massively parallel sequencing’. At this scale, it is possible to determine sequence from all protein coding regions of the genome (the exome), the whole genome, or even a subset of genes as part of a small collection of DNA molecules. As a result of this scale change, second-generation sequencing has become the predominant method for diagnostic sequencing in recent years.

A particularly novel approach to converting a pool of template into sequence-able material (known as a ‘library’ of templates), is the process of simultaneously fragmenting template molecules whilst adding the exogenous adaptors to their ends by use of an engineered Tn5 transposase bound to the exogenous adaptors (Nextera transposome, Illumina, San Diego, CA, USA [13]). This generates templates of a uniform size for second-generation sequencing, and ensures that the sequencing primer binding site is incorporated into the terminal regions of each template molecule, through a process known as tagmentation. This approach was utilized in conjunction with long-range PCR to enable large contiguous regions of COL2A1 to be made into a sequence-ready library for second-generation sequencing.

Exploiting the tagmentation of long-range PCR amplification products enabled a smaller number of amplicons to cover the entire gene region (in the case of COL2A1), and significant proportions of the intronic regions (in the case of COL11A1). The 33kb contiguous region of COL2A1 could be amplified in 12 reactions as opposed to the 46 required for capillary sequencing, for example, affording a significant increase in throughput. By multiplexing such an approach, this could be reduced to two amplification reactions, to tile sequence data across the entire gene, including all introns (Appendix B).

Alternative approaches to generating sequencing libraries based on amplicons include capture-based enrichment prior to sequencing. This approach uses magnetically labelled exogenous DNA and RNA sequences (probes) that are hybridized to the library prior to sequencing. This enables genes of interest to be enriched from a pool of library molecules, reducing the complexity of the subsequent library, and enabling targeted sequencing. Such approaches are suitable for sequencing large numbers of gene simultaneously, and can enrich a library constructed from a whole genome to generate sequences from solely protein-coding exons that are present. However, deep intronic regions distal to the conserved splice donor and acceptor sites are rarely represented in such approaches, due to the presence of repeat sequences and regions of cross-homology within those regions that decrease the selective enrichment capacity of the approach.

Such regions are commonly missing from sequence data, despite an appreciation for the clinical consequences of variants in these regions [14,15]. Intronic variants outside the conserved AG and GT dinucleotides of the acceptor and donor splice sites are frequently classified as being of unknown clinical significance due to the scarcity of intronic data in population databases of variants (gnomAD, https://gnomad.broadinstitute.org (accessed on 1 January 2022). A key component of the U.K. Stickler’s higher specialist service is the ability to ascertain the functional consequences of such variants on pre-mRNA splicing, using an in vitro functional assay [15]. This has demonstrated that the Stickler syndrome phenotype can be modified by variations in pre-mRNA splicing, leading to haploinsufficiency or dominant negative effects as a consequence [16].

## 7. Future Diagnostic Developments

Second-generation sequencing has matured steadily over the last decade, with Illumina and Thermo Fisher becoming the main suppliers of sequencing technology incorporated into diagnostic services. Future technological developments will include real-time sequencing from single-template molecules, also known as third-generation sequencing. Companies such as Oxford Nanopore and PacBio can offer significantly longer read lengths compared with first-generation and current second-generation sequencing using these approaches. Although this has enabled improvements in structural variation detection, the quality of sequence data generated from single molecules is not yet on par with Illumina’s sequencing by synthesis (SBS) approach. Whilst third-generation approaches continue to mature, so too do current second-generation offerings. Techniques have been developed to phase variants over long genomic distances despite utilizing short read sequencing technologies (www.10xgenomics.com, accessed on 1 January 2022); challenging templates, including triplet repeat regions and insertion/deletion events, can be sequenced and analysed (Expansion Hunter, Illumina San Diego, CA, USA). Whole-genome second-generation sequencing is now an affordable option in diagnostic service for constitutional and acquired genetics, and an ever-expanding number of whole-genome datasets are available for model organisms (https://www.ncbi.nlm.nih.gov/genome/, accessed on 1 January 2022). The validation of whole-genome sequencing from dried blood spots [17] affords a tantalizing insight into diagnostic genetics in the near future.

## Data Availability

Not applicable.

## Appendix A

Amplification primers and MasterAmp buffer (Cambio, Cambridge, UK) for first-generation sequencing. M13 sequence tag locations are shown in the primer name. PCR conditions: 95 °C, 5 min, followed by 33 cycles of 95 °C, 1 min, 60 °C, 1 min, 72 °C, 1 min, followed by 72 °C, 5 min.

**Primer Name**	**Sequence**	**Buffer**
COL2A1_PROM_F*M13F	cacccttcccgcctgtggtcagag	D
COL2A1_PROM_R	gtgcctaagtcggcgcgctacgac	
COL2A1_1_F*M13F	atgagggcgcggtagagac	E
COL2A1_1_R	gtgcctaagtcggcgcgctacgac	
COL2A1_2_F*M13F	tatgtccaggtggccccagcctac	E
COL2A1_2_R	ctgtccttcatgtgtcttggaagc	
COL2A1_3_F*M13F	gtggccctaacaccccaacagagg	E
COL2A1_3_R	cagcccctgtgttgaggtaccctc	
COL2A1_4_5_F*M13F	gagggtacctcaacacaggggctg	D
COL2A1_4_5_R	atgacagcaaggccaggagcctgc	
COL2A1_6_7_F*M13F	tctttctccgtcccttcctcgctg	D
COL2A1_6_7_R	cccttagcaccacagtctcatgcc	
COL2A1_8_F*M13F	cccttccagtgaaatgattttgcc	E
COL2A1_8_R	agaggttgtcagactctctggctc	
COL2A1_9_10_F*M13F	tgagagggagcagccactctaggcc	D
COL2A1_9_10_R	ctgggcagagcctgggagggacagc	
COL2A1_11_F*M13F	cccaatgtggcaaggaccaccagg	D
COL2A1_11_R	gtgcctccctgtcactccccaagc	
COL2A1_12_F	gccctctggggtgcccccactatgc	D
COL2A1_12_R*M13F	actgcccagcctccctcatgagag	
COL2A1_14_15_F	gaggccctcctgcagcccagggcag	D
COL2A1_14_15_R*M13F	atgaactttgcacaaagggagctc	
COL2A1_13_14_F*M13F	gaggccctcctgcagcccagggcag	D
COL2A1_13_14_R	atgaactttgcacaaagggagctc	
COL2A1_16_F	atcctggctagtcaaggagccagc	E
COL2A1_16_R*M13F	actgtgcagactcagcctgggaag	
COL2A1_17_F*M13F	gtgtgtccttcgttttctgtaagg	B
COL2A1_17_R	tgttgagggagcaatgagcaaggg	
COL2A1_18_F	gtgtgtccttcgttttctgtaagg	B
COL2A1_18_R*M13F	tgttgagggagcaatgagcaaggg	
COL2A1_19_F*M13F	gggtgcatgtgcataatttagtgc	A
COL2A1_19_R	cccacaactgtcagagcaaagtac	
COL2A1_20_21_F*M13F	ttcattctggcccaatgcctgtcc	D
COL2A1_20_21_R	ggtggtgggtcagtggggctgagg	
COL2A1_21_22_F	ttcattctggcccaatgcctgtcc	D
COL2A1_21_22_R*M13F	ggtggtgggtcagtggggctgagg	
COL2A1_23_F*M13F	ttcatactctgagtcgaggcttgc	D
COL2A1_23_R	gactatttcatgtcagtctggtgg	
COL2A1_24_25_F*M13F	ccaccagactgacatgaaatagtc	B
COL2A1_24_25_R	atctctcttttcccttgcttcccc	
COL2A1_25_26_F	ccaccagactgacatgaaatagtc	B
COL2A1_25_26_R*M13F	atctctcttttcccttgcttcccc	
COL2A1_27_F*M13F	tgggtgtgatgtggtcaatcctag	E
COL2A1_27_R	cccaaatcacatacagacccccac	
COL2A1_28_F*M13F	ggcctcagtccctgcagcccgctc	D
COL2A1_28_R	cccacattcacatctgtcagctcc	
COL2A1_29_F*M13F	tgtggaaatggagctcagctgggg	A
COL2A1_29_R	ctccaccaatgtgggtccacacag	
COL2A1_30_31_F*M13F	ctactagctgtggctctcagggtc	D
COL2A1_30_31_R	gggaggtggggaaaggagcaggag	
COL2A1_32_33_F*M13F	gagtgatattcagccctgctgtgg	D
COL2A1_32_33_R	tcattcctcctgagcccgctcctc	
COL2A1_34_F	agaggagcgggctcaggaggaatg	D
COL2A1_34_R*M13F	ctaacagaaaccttcatcaccagg	
COL2A1_35_36_F*M13F	gctttccctagcaccccagcctgg	D
COL2A1_35_36_R	cgcctttggcaggagataagaagg	
COL2A1_37_F*M13F	caaatgcactttgccctctcccac	E
COL2A1_37_R	acaagctccgatgcccgagggtgc	
COL2A1_38_F	caaatgcactttgccctctcccac	E
COL2A1_38_R*M13F	acaagctccgatgcccgagggtgc	
COL2A1_39_F*M13F	tcccgcctccatactaatagaacc	E
COL2A1_39_R	cacagcccacatgccacatggaag	
COL2A1_40_F*M13F	agccagaaccaagctgctgatctc	G
COL2A1_40_R	ttaggctggggaccaacgcagggc	
COL2A1_41_F*M13F	tccataccaggctctgagaccacc	G
COL2A1_41_R	gaaggccagcctggagctctccag	
COL2A1_42F_F*M13F	aagcccccagagaggaaactgctg	i
COL2A1_42F_R	ctgctccctcctaccccatgc	
COL2A1_42R_F	aagcccccagagaggaaactgctg	i
COL2A1_42R_R*M13F	ctgctccctcctaccccatgc	
COL2A1_43_F*M13F	agctcacagagcatggggtagg	D
COL2A1_43_R	tgacccagcacagagactcacagg	
COL2A1_44_F	agctcacagagcatggggtagg	D
COL2A1_44_R*M13F	tgacccagcacagagactcacagg	
COL2A1_45_F*M13F	ggcctgggcttctgagaggggctg	A
COL2A1_45_R	gtccttctaggctgagatgagact	
COL2A1_46_47_F*M13F	agtctcatctcagcctagaaggac	A
COL2A1_46_47_R	ccacccaagctgaggaatccccgg	
COL2A1_48_F*M13F	gctgggagggcagccagcctccag	D
COL2A1_48_R	cccagaagcagcagcatttccctc	
COL2A1_49_F	gctgggagggcagccagcctccag	D
COL2A1_49_R*M13F	cccagaagcagcagcatttccctc	
COL2A1_50_F*M13F	gagtggctggtgctatcaggacag	E
COL2A1_50_R	tgccctaaaagaggccctgagc	
COL2A1_51_F*M13F	gagggacactctagtacattctag	A
COL2A1_51_R	caggggccagggctgcagcttctc	
COL2A1_52_F*M13F	tctgtctctttcagtcaggcctgg	A
COL2A1_52_R	tttccctcctctcaagcccaacag	
COL2A1_53_F*M13F	tcctctgagcttgctccactcctgg	E
COL2A1_53_R	gccgcgggccaaccctcagccctg	
COL2A1_54_F*M13F	ttgttcagttttgggcttctgggc	D
COL2A1_54_R	agagtgactgagattggaaagtac	

## Appendix B

Amplification primers for second generation sequencing using SequalPrep Long range PCR (Thermo Fisher, Waltham, MA, USA). PCR conditions; 94 °C 2 min, 10 cycles of 94 °C, 10 s, 60 °C, 30 s, 68 °C, 8 min, followed by 25 cycles of 94 °C, 10 s, 60 °C, 30 s, 68 °C, 8 min (plus an additional 20 s per cycle) followed by 72 °C, 10 min.

**Primer Name**	**Sequence**
COL2A1_Prom-F	cacccttcccgcctgtggtcagag
COL2A1_Prom-R	ctgtccttcatgtgtcttggaagc
COL2A1_2-10_F	tatgtccaggtggccccagcctac
COL2A1_2-10_R	ctggtggtccttgccacatt
COL2A1_9-15_F	ggggagtgggaaatgagagg
COL2A1_9-15_R	atgaactttgcacaaagggagctc
COL2A1_13-17_F	gaggccctcctgcagcccagggcag
COL2A1_13-17_R	cagagtgctgctgtggttgc
COL2A1_17-22_F	cgccatcctcgtgctctgc
COL2A1_17-22_R	ggtggtgggtcagtggggctgagg
COL2A1_19-26_F	gggtgcatgtgcataatttagtgc
COL2A1_19-26_R	cccagtgcctaccatctaccc
COL2A1_24-31_F	ccaccagactgacatgaaatagtc
COL2A1_24-31_R	gggaggtggggaaaggagcaggag
COL2A1_30-36_F	ctactagctgtggctctcagggtc
COL2A1_30-36_R	cgcctttggcaggagataagaagg
COL2A1_35-41_F	gctttccctagcaccccagcctgg
COL2A1_35-41_R	gaaggccagcctggagctctccag
COL2A1_41-45_F	tccataccaggctctgagaccacc
COL2A1_41-45_R	gtccttctaggctgagatgagact
COL2A1_45-50_F	ggcctgggcttctgagaggggctg
COL2A1_45-50_R	tgccctaaaagaggccctgagc
COL2A1_50-54_F	gagtggctggtgctatcaggacag
COL2A1_50-54_R	agagtgactgagattggaaagtac
